# Understanding Adolescents’ Experiences With Menstrual Pain to Inform the User-Centered Design of a Mindfulness-Based App: Mixed Methods Investigation Study

**DOI:** 10.2196/54658

**Published:** 2024-04-08

**Authors:** Michelle M Gagnon, Alexandra R Brilz, Nicole M Alberts, Jennifer L Gordon, Tracie L Risling, Jennifer N Stinson

**Affiliations:** 1 Department of Psychology and Health Studies University of Saskatchewan Saskatoon, SK Canada; 2 Department of Psychology Concordia University Montreal, QC Canada; 3 Department of Psychology University of Regina Regina, SK Canada; 4 Faculty of Nursing University of Calgary Calgary, AB Canada; 5 Lawrence S. Bloomberg Faculty of Nursing University of Toronto Toronto, ON Canada; 6 Child Health Evaluative Sciences, Research Institute Hospital for Sick Children Toronto, ON Canada

**Keywords:** adolescent health, endometriosis, pain management, biopsychosocial, women’s health, dysmenorrhea, thematic analysis, mHealth, mobile health, app, apps, applications, applications, attitude, attitudes, opinion, opinion, perception, perceptions, perspective, perspectives, interest, intent, intention, survey, surveys, focus group, focus groups, content analysis, mindfulness, meditation, menstrual, menstruation, experience, experiences, pain, youth, adolescent, adolescents, teen, teens, teenager, teenagers

## Abstract

**Background:**

Digital interventions are increasingly popular for the provision of nonpharmacological pain interventions, but few exist for adolescents with menstrual pain. User-centered design involves incorporating users across phases of digital health intervention design, development, and implementation and leads to improved user engagement and outcomes. A needs assessment is the first step of this approach.

**Objective:**

The goal of this study was to conduct a needs assessment to understand menstrual pain management needs and preferences and mindfulness experiences, preferences, and knowledge of adolescents with menstrual pain to inform the future development of an app for managing menstrual pain.

**Methods:**

We used an explanatory sequential mixed method design that included a survey followed by focus groups. Adolescents aged 13-17 years completed a survey (n=111) and participated in focus groups (n=16). Data were analyzed using descriptive statistics and thematic content analysis and synthesized to provide specific recommendations based on adolescent responses.

**Results:**

Adolescents (n=111) who completed the survey reported a moderate understanding of mindfulness and menstrual pain. Over three-quarters (n=87, 78%) of participants practiced some form of mindfulness and 87% (n=97) of survey participants used nonpharmacological pain management strategies. Teens had a moderate perception that mindfulness could help their menstrual pain (mean 4.51/10, SD 2.45, with higher scores suggesting more interest). Themes were generated related to mindfulness experiences, menstrual pain knowledge and experiences, and app functionality. These themes underscored adolescents’ need for continued support and flexible access to mindfulness activities; their awareness of multiple influences to pain, with potential for further education in this area; and the need for menstrual pain–specific content, along with content relevant to typical day-to-day experiences of adolescents.

**Conclusions:**

Adolescents with menstrual pain have an interest in using a mindfulness app for pain but have unique needs that need to be addressed to ensure app engagement and relevance for this population. Concrete recommendations for future app development are provided.

## Introduction

Dysmenorrhea, or menstrual pain, is experienced by over 90% of adolescents who menstruate and has been associated with mental health symptoms, nonsuicidal self-injury, and decreased quality of life [1-3]. For a quarter of these youth, the severity of their pain prevents them from engaging in daily activities and contributes to school absenteeism [4]. Increasingly, it is recognized that persistent, unmanaged dysmenorrhea may lead to the development of chronic pain [5,6]. Despite the impacts of dysmenorrhea on adolescent well-being, there are few accessible, evidence-based treatment options available for adolescents. Prevailing interventions are primarily pharmacological, which is not always a good fit given potential side effects and barriers to accessing care [7,8]. There is a need for accessible and effective interventions for adolescents with dysmenorrhea.

Digital health interventions, such as app-based interventions, are increasing in popularity, particularly given their ability to reduce barriers to accessing health-related services [9,10]. Recent estimates show that 95% to 98% of youth and young adults in Canada and the United States have a smartphone [11,12]; the ubiquity of smartphone access suggests that an app-based intervention maybe a viable option for adolescents with menstrual pain in Western populations. Although there is a plethora of apps related to menstruation [13,14], only a fraction of these apps contains pain-related content, and the accuracy and quality of this content are low [15,16]. Importantly, few of the available menstrual pain apps are designed specifically for adolescents [16] who may have different menstrual knowledge [3], menstrual cycle characteristics [17], and app use preferences than adults. Consequently, adolescents may benefit from an app designed specifically for their age group [10,18,19].

User-centered design is a digital intervention design approach that involves collaboration with end users (eg, patients and parents) throughout all stages of app development and implementation. User-centered design in the development of health-based apps has been shown to increase satisfaction, mitigate barriers to engagement, and improve user adoption [20-23]. In the user-centered design, the end user is included in the development process through the use of focus groups and surveys, participatory design sessions, and usability testing [24]. This multistep approach begins with a user needs assessment [21,25], which is integral in identifying and prioritizing the needs of the end user, in this instance, adolescents with menstrual pain.

To our knowledge, there are no apps developed with and for adolescents that incorporate both user-centered design and evidence-based content for the management of dysmenorrhea. Evidence-based nonpharmacological therapies such as cognitive behavioral therapy and mindfulness-based interventions have been applied as treatments for chronic and specific pain-related disorders [26,27]. As a first step in filling the gap in access to nonpharmacological interventions, this study focused on adolescents’ perception of mindfulness as a potential intervention for dysmenorrhea.

Mindfulness-based interventions are rooted in theories of mindfulness and focus on changing one’s relationship with pain and suffering [28]. Research suggests that mindfulness-based interventions may be beneficial in adolescent populations for a range of concerns [29-31] and have demonstrated potential in reducing pain and improving pain acceptance and ability to cope in youth with chronic pain [32]. The aspects of mindfulness in combination with other therapeutic approaches have also been successfully integrated into face-to-face treatments for adults with dysmenorrhea [33]. In this investigation, we focused on determining adolescents’ interest in mindfulness over other interventions for several reasons. First, past research suggests that many youth report positive experiences with mindfulness programs and the impact of mindfulness skills on their day-to-day lives [34-36]. Mindfulness-based interventions also have the potential to lend themselves better to younger teens over approaches that may require adaptation based on cognitive skills [31,37-39]. Additionally, mindfulness strategies (eg, meditations) may be more easily applied by youth in an app-based platform without therapist guidance than other interventions [40,41], which is important given the high prevalence of dysmenorrhea yet limited availability of nonpharmacological treatment options [17,42].

This study reports the first step in the user-centered development of a self-management, mindfulness-based app for teens with dysmenorrhea, that is, a needs assessment. Although digital modalities for the delivery of mindfulness-based interventions, such as smartphone apps, may be a promising means through which pain interventions may be offered, understanding whether teens are interested in mindfulness-based strategies for their pain and what type of content would be valuable to them in an app is essential. The goals of this needs assessment were to identify (1) mindfulness knowledge, experiences, and preferences; (2) menstrual pain management knowledge, experiences, and preferences; and (3) app use experiences and preferences among Canadian adolescents who experience dysmenorrhea. Using a 2-phased approach that relied on both administering a survey (phase 1) and conducting focus groups (phase 2), adolescent views were gained to inform future app development. The findings of this investigation will be used to engage in subsequent steps of the user-centered design process, including usability testing and pilot testing of the resulting app.

## Methods

### Research Design Overview

An explanatory sequential mixed methods design was selected for this investigation [43]. In this approach, quantitative data are collected first and are followed by the collection of qualitative data to gain a deeper understanding of quantitative findings. In phase 1 of our investigation, descriptive quantitative data and narrative responses were collected via a web-based survey. These findings of this survey are reported in accordance with the CHERRIES checklist (Checklist for Reporting Results of Internet E-Surveys) [44] (Multimedia Appendix 1). Subsequently, phase 2 involved focus groups to gain further insight into the experiences of adolescents described in phase 1. Qualitative findings from the focus group responses are reported in accordance with the COREQ (Consolidated Criteria for Reporting Qualitative Research) checklist [45] (Multimedia Appendix 2).

### Participants

A convenience sample of participants aged 13 to 17 years was recruited between January and July 2022 to participate in a web-based survey (phase 1) and a focus group (phase 2). To be eligible, adolescents were required to have had their first period, experience pain with their period, and live in Canada. Adolescents who were aged 13 to 15 years were required to have consent from a parent or legal guardian to participate in the survey (note that for simplicity, we refer to “parent” to reflect all guardians who provided consent from hereon). All participants (ie, 13-17 years of age) were required to have parental consent to participate in the focus group.

### Ethical Considerations

This study was approved by the University of Saskatchewan Research Ethics Board (BEH 3013). Survey responses from participants aged 16-17 were anonymous. Participants aged 13-15 were known to the research team, but did not report identifying information in the survey and rather entered a participant ID to ensure that survey responses were from participants who had appropriate parental consent. Due to the nature of the focus groups, anonymity was not possible, but the importance of maintaining other participants' confidentiality was discussed prior to beginning each focus group. Survey participants were entered into a draw for 1 of 5 CAD $50 (approximately US $36.89) Amazon gift cards. Focus group participants received the choice of a CAD $50 honorarium or a CAD $50 Amazon gift card (approximately US $36.89) for their participation.

### Measures

#### Needs Assessment Questionnaire

The 27-item needs assessment questionnaire was developed to gather information about adolescents’ (1) phone use patterns and preferences (5 items); (2) mindfulness knowledge and experiences (6 items); (3) pain knowledge and impact of menstruation on daily functioning (5 items); (4) use of existing menstrual and mindfulness apps (1 item); and (5) perceived barriers and facilitators of, interests in, and recommendations for a mindfulness app for adolescents with menstrual pain (6 items). Additionally, the questionnaire queried demographic information (4 items), including age, sex assigned at birth, gender identity, and ethnic or racial background. Question formats throughout the survey included yes or no questions (eg, “have you ever practiced mindfulness or meditated?”), multiple-choice responses (eg, “when do you experience the most pain or physical discomfort with your period [select all that apply]?”), 11-point numeric rating scales (eg, “what would you rate your level of understanding of what mindfulness is?”; 0=no understanding and 10=complete understanding), and open-ended questions (eg, “what would motivate you or help you to use the app to use mindfulness when you are experiencing period pain?”). The measure was developed by the first author (MMG) and reviewed by all coauthors and study research assistants.

#### Focus Group Interview Guide

A 10-question semistructured interview guide was developed by our multidisciplinary team and comprised questions that built upon the information gathered in the needs assessment survey. Participants were asked about their experiences with pain, including their understanding of contributors to their pain, their experiences with mindfulness, their experience with menstrual apps, their interest in using a mindfulness app for period pain, their perception of challenges and motivators for using health apps, and any suggestions to inform future app development.

### Procedure

#### Phase 1: Web-Based Survey

Adolescents were the primary participants of this study; however, to increase enrollment, the parents of adolescents were also targeted throughout the recruitment and involved in the enrollment phase to provide consent for their adolescent to participate where needed. Participants were recruited using Facebook and Instagram advertisements posted on the research team’s Facebook and Instagram accounts targeting adolescents and parents of adolescents.

Participants aged 16 and 17 years accessed the survey via a link provided in recruitment materials. Prior to gaining access to the survey, participants were required to review the consent form outlining the rationale for the study, the duration of the survey, data storage and security, confidentiality measures, and their right to withdraw. After reviewing the consent form, participants provided their informed consent by proceeding to the next page of the survey. For participants aged 13-15 years, the interested adolescent or their parent completed a web-based form to indicate the adolescent’s interest in the study. A research assistant contacted the parents of each adolescent to confirm the adolescent’s eligibility and obtain parental consent. Adolescent assent was also obtained. Following receipt of consent and assent, the research assistant sent the adolescent a link to the web-based questionnaire.

The survey duration was approximately 10-20 minutes, and the survey was conducted via SurveyMonkey. All survey questions were voluntary and presented across 6 pages with backtracking enabled. Question randomization was not used, and no questions were conditionally displayed. The survey was distributed via 2 survey links: an open survey for adolescents 16-17 years, where IP addresses were used to identify unique survey respondents and prevent multiple, same-person responses, and a closed survey for adolescents 13-15 years, where the link was provided directly to participants by the researchers. Participants had the option of providing their email to be contacted regarding the focus group, entered into the draw for the gift card, or both.

#### Phase 2: Web-Based Focus Groups

Participants were recruited using Facebook and Instagram advertisements targeting adolescents and parents of adolescents posted on the research team’s Facebook account. Additionally, phase-1 adolescents interested in participating in a focus group were able to indicate their interest at the end of the survey. Interested participants were contacted by a research assistant to schedule a consent and assent call via Zoom (Zoom Technologies Inc) with the adolescent and their parents. During the call, the research assistant reviewed the purposes of the study using a shared PowerPoint (Microsoft Corp) presentation. If the family remained interested in participating, the research assistant reviewed the consent and assent information with the family, enrolled the adolescent, and gathered demographic information.

Participants were enrolled in 1 of 3 groups based on their availability. Focus groups were conducted digitally via Zoom and were audio and video recorded to allow for later review. Each group had 4 to 7 participants and lasted up to 90 minutes. All researchers (ie, MMG, ARB, and a research assistant) involved in the focus groups were Canadian female individuals. Focus groups were led by 1 member of the research team and a second team member took field notes. Following the completion of the focus groups, audio from the groups were transcribed verbatim to allow for analysis.

### Analytic Plan

#### Overview

Demographic information from the survey and the focus group were analyzed using descriptive statistics. Across both phases of the sequential explanatory design, we used SPSS (IBM Corp) to calculate quantitative results, and Microsoft Excel was used for the organization of narrative data. Survey responses and focus group responses were analyzed separately, and results were grouped based on findings that addressed each of the study objectives (ie, mindfulness knowledge and experience, dysmenorrhea knowledge and experiences, and app use preferences). Narrative responses from open-ended survey questions and phase-2 focus groups were examined using an experiential realist framework. In an experiential framework, the analyses aim to explore participants’ understanding, while a realist framework aims to capture (rather than construct) the reality of participants within the data set [46]. This framework was chosen, given the study goal of understanding adolescents’ experiences as a means of proposing concrete recommendations for app development.

#### Analysis of Survey Responses

Closed-ended survey questions from the needs assessment survey were analyzed using descriptive statistics. Open-ended questions of the needs assessment survey questions were examined question by question. Codes were formulated from responses with no limit placed on how many codes could be developed. Developed codes were then examined for similarities and grouped into themes. Theme frequency was reported to understand how important ideas were to adolescents compared to other ideas [47].

#### Analysis of Focus Group Responses

Reflexive thematic analysis [46] was used for analysis of the focus group data. Following verbatim transcription of the focus group recordings, an inductive (ie, data-driven) approach to examining the data was taken, with a focus on the semantic level of meaning of responses. Codes were formulated within each question of the focus group interview by 1 member of the research team (MMG), again with no limit placed on the number of codes, and similar codes were grouped to formulate themes. Reflexive thematic analysis involves critically considering the researchers’ personal and professional experiences and biases that may impact data interpretation. The research team is interested in improving digital interventions, with backgrounds in nursing and psychology.

#### Synthesis of Qualitative and Quantitative Findings

To bring a comprehensive understanding of the ideas from each phase and research objective (ie, mindfulness, menstrual pain, and app preferences), the qualitative and quantitative findings were synthesized. The themes generated from focus groups were combined with survey findings related to mindfulness and dysmenorrhea knowledge and experiences and app use preferences to identify resulting needs for adolescents. Specific recommendations for future app development were then generated based on the combined results.

## Results

### Demographic Information

A total of 143 participants completed some portion of the survey and 108 (76%) completed the final page. Participants who had completed less than 80% of survey questions (n=32, 22%) were considered to have withdrawn and were removed, resulting in a final sample of 111 participants. Demographic characteristics of participants in both phases are summarized in Table 1.

**Table 1:** Demographic characteristics of survey participants (n=111) and focus group participants (n=16).

Characteristics		Survey participants	Focus group participants
**Age (years)**			
	n	109	16
	Mean (SD)	15.97 (1.21)	15.19 (1.52)
	Range	13-17	13-17
**Gender identity, n (%)**			
	Girl or woman	103 (93)	15 (94)
	Transgender	1 (1)	N/A^a^
	Other	7 (6)	1 (6)
**Racial or ethnic origin, n (%)^b^**			
	Arab	3 (3)	1 (6)
	Black	2 (2)	N/A
	Chinese	2 (2)	N/A
	Filipino	1 (1)	N/A
	First Nations	10 (9)	N/A
	Métis	4 (4)	1 (6)
	Inuit	1 (1)	N/A
	Latin American	3 (3)	N/A
	South Asian	3 (3)	3 (19)
	Southeast Asian	1 (1)	N/A
	White	93 (84)	13 (81)
	Other	1 (1)	1 (6)

^a^N/A: not applicable.

^b^Participants could provide a response in more than 1 category.

### Phone and App Use

Among survey participants (n=111), 87% (n=95) had an Apple device and 14% (n=15) had an Android device. Most participants did not have unlimited data (n=70, 63%) or were unsure (n=9, 8%). Most used apps included social networking apps (n=107, 96%), utility apps (n=82, 74%), entertainment apps (n=81, 73%), productivity apps (n=69, 62%), and gaming apps (n=63, 57%).

### Mindfulness Knowledge and Experiences

#### Survey Results

Nearly all survey participants (106/111, 95%) reported having heard of mindfulness. Participants reported a moderate understanding of mindfulness (mean 6.63, SD 2.32 out of 10; 0=no understanding and 10=complete understanding) and how to use mindfulness (mean 5.70, SD 2.59 out of 10; 0=no understanding; 10=complete understanding). Over three-quarters (87/111, 78%) of respondents reported having practiced mindfulness. The most common types of mindfulness activities used by 84 participants who reported any type of activity were meditation (n=57, 68%), yoga (n=53, 63%), breathing (n=32, 38%), music (n=17, 20%), movement (eg, walking; n=10, 12%), and art-based activities (eg, coloring; n=5, 6%). Among survey participants, 53 (48%) reported using meditation apps. YouTube (n=17, 32%), Spotify or other podcast apps (n=10, 19%), Headspace (n=8, 15%), and calm (n=7, 13%) were the most used meditation apps or websites.

#### Focus Group Results

##### Overview

Focus group participants were asked to discuss their experiences with mindfulness. Participants’ experiences largely occurred in school settings, through their parents, or in mindfulness-based extracurricular, such as yoga classes. Three themes were generated based on participants’ described experiences.

##### Theme 1: Discouraging School Experiences

Exposure to mindfulness at school was common and participants with this experience described challenges with learning mindfulness in this setting. Responses were categorized into 2 subthemes. The first subtheme related to *discontinued support.* Several participants reported that mindfulness was practiced throughout elementary and middle school but that in high school support from teachers in learning and practicing mindfulness stopped, making it difficult to maintain their mindfulness practice. This pattern of reduced support as students age was captured by a participant who described:

Before [in elementary school] it’d be every day we’d have something to do with [mindfulness]. Now [in high school] you kind of fend for yourself. You don’t get too much support anymore.

Participant 22, 13 years old

A second subtheme related to the school setting being an *unconducive environment* for mindfulness was generated. Participants described difficulty practicing mindfulness at school because of distractions in the classroom. They also described feeling self-conscious due to having their peers around or being distracted by noise, which interfered with their perception of how helpful the practice was:

When we did it at school...there was a lot of people talking...and it makes it really hard to concentrate. So, I don’t think it helps in large groups of people who don’t want to take it seriously.

Participant 42, 17 years old

##### Theme 2: Personalization Needed

Participants described a need for mindfulness activities to be interesting or personalized to the individual. The generality of mindfulness was identified as a barrier to its use:

I do agree that mindfulness is important and applies to everyone and what can help anyone in many ways. But it doesn’t necessarily seem all that inviting when it’s super general.

Participant 61, 16 years old

Many participants identified yoga as a way through which they practiced mindfulness. Participants noted that not all youth found sitting and meditating to be the most beneficial approach. These ideas were captured by a participant who stated:

Some people, especially as teenagers with emotions and energy and whatnot, might find it hard to relax. I know a lot of people who just can’t relax or [they] find it really hard to sit still and not talk, but I think mindfulness was a part of finding your way to do that. And if your way is movement or if your way is sitting there with your head down –mindfulness is finding a way that works for you in a way that you find beneficial.

Participant 124, 14 years old

##### Theme 3: Real-Time Use of Mindfulness Is Tricky

Integrating mindfulness into everyday life or sustaining a mindfulness practice was a challenge for youth. The challenges identified by youth included difficulty using mindfulness when it would be most needed, difficulty using strategies when busy, or the tendency to stop using mindfulness when there is no motivation to continue. A participant described the following experience trying to implement mindfulness on their own:

I think it did help in terms of lowering my heartrate and making me feel more calm and in the present. But I feel like sometimes when I try to incorporate it into my daily life it just never turns out right, because if I’m stressed, I can’t think of a way to calm myself down. It just doesn’t work for me that well.

Participant 1, 16 years old

The following quotation from a participant captured the challenges with sustaining the practice:

...because of COVID I had to stop going [to a yoga class] and now I’m so busy with schoolwork. So now [it’s] just pushed aside and [after COVID-19] mindfulness isn’t something I do once a week or something like that.

Participant 21, 14 years old

### Dysmenorrhea Knowledge and Experiences

#### Survey Results

On average, adolescents who completed the survey rated their knowledge of contributors to period pain and symptoms as moderate (mean 5.39, SD 2.30 out of 10; 0=no knowledge and 10=extremely knowledgeable). They also reported moderate levels of knowledge related to how to manage their menstrual pain (mean 5.78, SD 2.46 out of 10; 0=no knowledge and 10=extremely knowledgeable). Most survey participants (97/111, 87%) reported using nonpharmacological pain management strategies to manage menstrual symptoms, including hot water bottles or heating pads (83/111, 75%), rest or sleep (81/111, 73%), baths or showers (73/111, 66%), exercise (28/111, 25%), relaxation exercises (21/111, 19%), and mindfulness or meditation (16/111, 14%).

#### Focus Group Results

##### Overview

Focus group participants were asked to discuss their understanding of factors that contribute to their pain, which resulted in 2 themes.

##### Theme 1: Wavering Degree of Control Over Menstrual Pain

Participants described their ability to control pain as a variable across their menstrual cycle and impacted by environmental and individual factors. This theme led to 3 subthemes related to the participants’ experiences. The first subtheme related to how *context matters*. Participants described that the degree to which they felt comfortable in a situation affected their pain level. Being familiar and comfortable with those around them and being in low stress and calm situations were helpful in managing pain. Similarly, when participants felt in control of the situation, pain was easier to tolerate. This subtheme was captured by the following:

If you’re somewhere comfortable where you know you have the resources...you feel a lot more comfortable when you get pain. When you’re out in public or you have something to do that you need to do you’re like a lot more uncomfortable, and it can even be worse because you’re not like listening to your body.

Participant 124, 14 years old

The second subtheme related to *moments of helplessness* due to menstrual pain. Participants recognized that although pain was generally not constant, there were times in their lives when they felt a loss of control due to pain. This was reflected by 1 participant who stated:

I literally woke my parents up at night screaming because my cramps were so bad and I felt bad for screaming. But they hurt that much and that was to the point that one night my mom was like, ok, that’s it, you’re going on birth control. And I mean, it helps a bit. But, at the same time, I still get cramps and I’m nauseous, which makes me think maybe I should try a different birth control because in gym, if I go and do something, I get cramps just by doing stuff. And it’s really annoying because when I have to go sit off to the side.

Participant 143, 17 years old

The final subtheme was *pain management is challenging.* Within this subtheme, there were varying perspectives related to how medication and self-care could be helpful. Several participants acknowledged being aware of activities they could do to help with pain but struggled to be motivated to engage in them. This challenge was well reflected by a participant who stated:

...doing exercise helps, but when I’m in pain, to actually start doing it is really, really hard. So, most of the time I just don’t end up doing it because I don’t feel like getting up and actually starting. But if I were to start, it would probably be better.

Participant 127, 13 years old

Within this subtheme, several participants also described medications as either being the only strategy that helped or the only treatment option available to them:

I have really bad period pain to the point where I had to get an [intrauterine device], because that was the only thing that would stop it, and then I still had to get prescribed naproxen. So, I know...people say, that exercising helps with period pain, but for me, I find it makes it worse. I cannot really keep doing anything. And, well, nothing will make it really better. Meds would but that’s about it.

Participant 118, 16 years old

##### Theme 2: Mind-Pain Interaction

Participants identified a relationship between what went on in their minds and their menstrual pain. Several components of the “mind” influences were discussed by participants. For instance, they described being aware that engaging in another activity could help distract from the pain but that thinking about pain most often led to more pain:

I know for me when I have any type of pain, if I’m busy doing something all day or out with friends I don’t really notice it as much. And then I come home and I’m lying in bed and it usually hurts more when I’m thinking about it. Then [the pain] usually just doesn’t go away until I’m busy again or get myself watching a show or something, and then I just kind of forget about it.

Participant 42, 17 years old

Participants also believed that their mood, stress, or the anticipation of pain could increase pain. These ideas were captured in the following quote:

My mood definitely affects how it feels. When you’re at school and you’re getting stressed about something or you’re doing physical activity at school or even if it’s hanging out with...people who make you feel a certain way, whether that be stressed, annoyed, that sort of thing – it definitely aggravates how you feel. And then it aggravates how you’re feeling in regards to your period, your pain.

Participant 31, 15 years old

### Perceptions of Current and Potential App Use

#### Survey Results

Nearly two-thirds (70/111, 63%) of survey participants reported using a website or app to track their period, with Flo (34/111, 31%) and Clue (21/111, 19%) being the most common. Participants had a moderate perception that mindfulness could help cope with menstrual periods (mean 4.51, SD 2.45 out of 10; 0=not at all helpful and 10=extremely helpful). Approximately one-third (36/107, 34%) of participants provided a rating of 3 or lower out of 10 that mindfulness could be helpful, 43% (46/107) provided a rating from 4 out of 10 to 6 out of 10, and nearly a quarter (25/107, 23%) of participants provided a rating of 7 out of 10 or higher (Multimedia Appendix 3). Participants (n=111) indicated that if they were to use mindfulness during their period, they would likely use it 1-2 times (n=59, 53%) or 2-4 times (n=42, 38%). From a list of preferred app features provided to participants, adolescents were most interested in period tracking features, receiving feedback after logging pain and symptoms, and seeing insights into their mindfulness use (Table 2).

**Table 2:** Survey participants’ endorsement of each proposed app feature.

App feature^a^	Values (n=110), n (%)
Charts that track your pain from period to period	99 (89)
Receiving feedback after logging your menstrual pain (eg, recommendations to help manage pain)	98 (88)
Seeing how you are doing over time with practicing mindfulness (eg, tracking chart and number of minutes practiced)	75 (68)
Notifications reminding you to track your period	75 (68)
Charts that track other aspects of your period from period to period	67 (60)
Receiving positive feedback after logging your mindfulness practice (eg, way to go! and congratulations!)	66 (60)
Notifications reminding you to practice mindfulness	47 (42)
Sharing how you have been doing on the app with family and friends	10 (9)
Other	13 (12)

^a^Participants could provide a response in more than 1 category.

Survey participants were asked to indicate what would motivate and deter them from using an app for mindfulness when experiencing period pain. The themes from survey participant responses and the frequency of these responses are summarized in Table 3. Reminders to use the app, the app being effective in reducing their pain or increasing knowledge about periods, and the app being engaging were identified as essential motivators. Common barriers included the app not being free, being difficult to use, or having ads.

**Table 3:** Barriers and facilitators to app use among survey participants.

Barriers and facilitators		Values (n=111), n (%)
**Themes related to facilitators to use^a^**		
	Reminders	32 (30)
	Effective	16 (15)
	Engaging or rewards	14 (13)
	Encouraging or inspiring messaging	11 (10)
	Design or aesthetic	8 (8)
	Research evidence	8 (8)
	Charts or tracking	6 (6)
	Feedback	5 (5)
	Other	6 (6)
**Themes related to barriers to use^a^**		
	Not free	22 (22)
	Difficult to use (eg, complicated, glitches, or lags)	21 (21)
	Ads	19 (19)
	Too many notifications	15 (15)
	Visually unappealing or poor design	14 (14)
	Not helpful	9 (9)

^a^Participants’ responses were categorized into more than 1 theme, when applicable.

#### Focus Group Results

##### Overview

Focus group participants were asked to consider how or when they could see themselves using a mindfulness app to help with their period pain. Two themes were generated.

##### Theme 1: Manage Painful Parts of Period

Adolescents were most willing to consider using an app during the painful parts of their period. Several indicated they might not be motivated to use the app during parts of their cycle when they did not experience pain.

I would probably use it the first three days when I start [my period], because that's when the pain is the worst. But then after that it’s just fine. So, then I'm good.

Participant 81, 14 years old

Participants also noted that an app that helped with the management of severe pain would be beneficial if it provided tools that could be used when not feeling able to move due to pain. This was reflected by the following response:

I just think when I see things like “go out and take a hike, breathe some nature,” I'm not feeling very obligated to do that when I'm in my sweatpants and my hair's a mess. I don't really want to do that. But, you know, little things that I can do from the comfort of my bathroom floor would totally help me out in that second and third day during my cycle.

Participant 119, 17 years old

##### Theme 2: Navigate Emotions During Menstrual Cycle

Participants frequently mentioned that emotions that occurred around the time of their period could lead to challenges in their lives. Participants discussed that having tools to manage these emotions would be useful and that they would be motivated to use an app that addressed aspects of their lives beyond pain. For instance, 1 adolescent stated:

If it was paired with things I can do to manage those extreme emotions, that would be so incredibly helpful. That would really help me out because paired on top of teenage hormones, mental illness, and then that period pain and that irritability, things get wild. So, it would really be helpful if I had something like that in place.

Participant 119, 17 years old

### Synthesis of Results: A Summary Model

**Figure 1:**
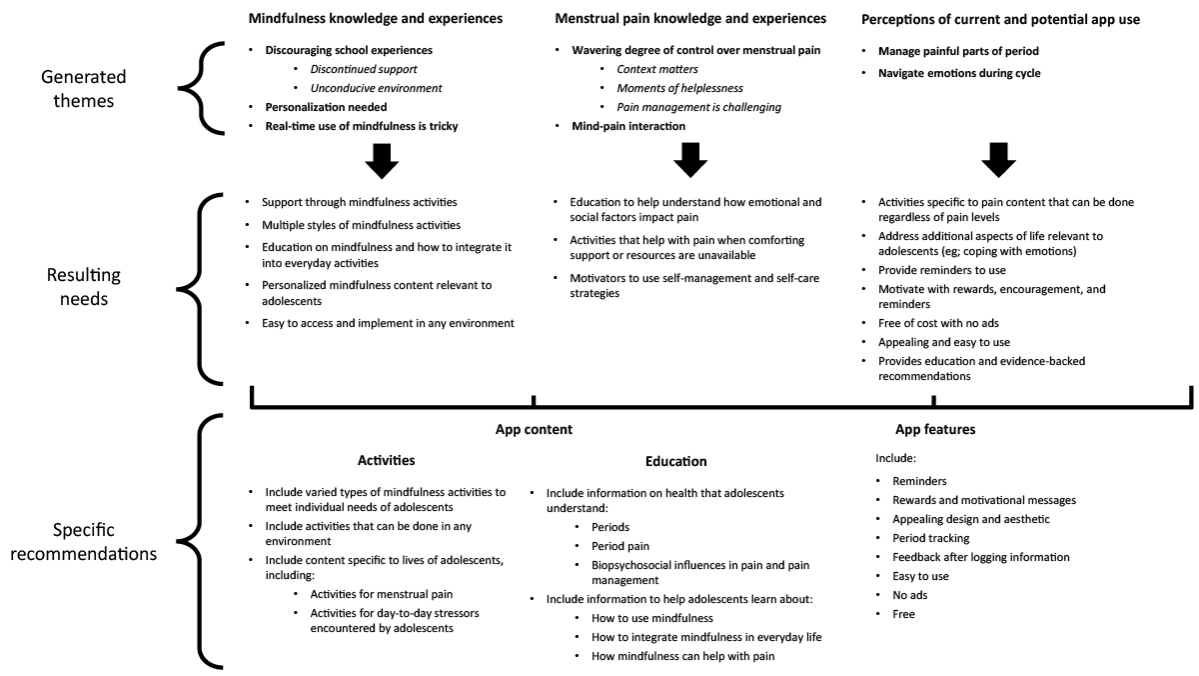
Summary model of survey and focus group findings. Generated themes and survey responses were transformed into resulting needs. From the needs, specific recommendations are made for content and features of a future app.

The findings from the data synthesis process are summarized in Figure 1. The 7 themes and 5 subthemes generated throughout the focus group analysis were merged with survey findings to result in 5 needs related to mindfulness, 3 needs related to menstrual pain, and 7 needs related to app use in menstruation (Figure 1). This process of connecting findings across phases allowed for the development of specific recommendations for future app development. Recommendations were divided into content recommendations and design or app feature recommendations.

Regarding content recommendations, adolescents wanted information specific to dysmenorrhea, along with activities and information relevant to the experiences and emotions of adolescent life. To counteract inconsistent school support, the app should provide easy access to ongoing support and be usable in any environment. Education content needs to improve understanding of periods, pain, and emotions; evidence-based information is important to youth. Education content needs to outline how mindfulness can help with pain and day-to-day experiences. Recommendations related to features include continuous access, being visually appealing and easy to use, having motivators or rewards built in, having reminders, and that the app be ad-free and of no cost.

## Discussion

We examined the specific needs and experiences of adolescents to inform the future development of a mindfulness-based, self-management app for dysmenorrhea. Overall, there was general agreement that mindfulness could be helpful for dysmenorrhea. In line with findings from other researchers [48], many adolescents engaged in some form of nonpharmacological pain management. However, participants reported experiencing pain management challenges and feelings of helplessness around pain, and this was the case even among some participants who used pharmacological options. This is not surprising given that prescribed medications are estimated to only work for a quarter of individuals who use them [8]. Only a small percentage of this survey sample used meditation or mindfulness to manage their menstrual symptoms. Despite low use of mindfulness specifically for menstrual pain, most youth surveyed had used apps or websites to access mindfulness activities, suggesting interest in these approaches for developing mindfulness skills.

Over half of the survey participants had experience with mindfulness, with exposure in school being common, albeit suboptimal. This is consistent with research showing that school-based mindfulness programs may not be effective [49]. Focus group participants provided insight into the reasons for this, including the environment being distracting and the inconsistent support that occurs in school settings. These findings suggest that future app-based interventions need to provide easy and continuous access to guided mindfulness activities. This could be achieved through ongoing access to the app and supportive information in the app, or by the inclusion of some form of app-based mindfulness coaching (eg, access to professional or peer support).

The need for a personalized experience was evident across themes. In school settings, focus group participants were dissatisfied with the generality of mindfulness programs and desired activities tailored to their personal preferences. Difficulty being still and staying engaged with meditations are common for youth [50], and many survey and focus group participants identified active forms of mindfulness, such as yoga, as beneficial. Adolescents also desired a variety of mindfulness activities based on their pain level; mindfulness activities that could be performed lying down when experiencing incapacitating pain were requested by some participants, which contrasted with other youth who preferred movement-based mindfulness activities for when the pain was tolerable. Overall, these diverse needs illustrate that app-based interventions must offer the ability to select from a variety of activities based on personal preference and pain level.

The need for personalization in relation to app content was also identified. Focus group participants voiced the desire for an app that addresses the unique needs of youth, including navigating difficult emotions that may occur throughout adolescence. In line with well-established research across pain conditions [51], adolescents noted increased pain when experiencing strong emotions, as well as pain being exacerbated by social or situational factors. Focus group participants’ desire for the inclusion of mindfulness activities that address mental, social, and physical aspects of their day-to-day lives is an important finding of this investigation.

Participant responses also pointed to a need for educational content relevant to early menstruators. Survey participants indicated that evidence-based information explaining how mindfulness may improve pain would motivate them to use the app. Although a general understanding of dysmenorrhea was reported by participants, previous research has found that most youth desire more information about menstrual health [3]. Education programs offered to young women to improve knowledge of dysmenorrhea are effective and increase knowledge and the likelihood of seeking professional help [52].

Across our findings, the importance of a biopsychosocial focus to any future app is evident from the participants’ indication that an app needs to address various aspects of their lives and participants’ understanding that their pain is affected by more than just their physical experiences. Biopsychosocial considerations are often overlooked in the context of dysmenorrhea [53]; yet, psychoeducation about the biopsychosocial nature of pain is an integral piece to pain management programs [51]. Such educational information may help adolescents engage with psychological approaches and understand how they may apply in the context of period-related pain. Psychoeducation on mindfulness and how it might affect pain would also be relevant in addition to mindfulness activities.

Overall, our findings reflect the relevance of conducting a needs assessment as adolescents provided insightful information into the needs of a future app, which may have been overlooked. In line with a user-centered design, future phases of app development should engage youth to evaluate app content and design to improve the engagement of youth in the app. Nevertheless, this study is not without its limitations. Our sample size is small as the purpose was to understand users’ needs rather than to gain an understanding of the prevalence of experiences. Participants were predominantly White and identified as a woman or girl. Adolescents who participated may have had more interest in or knowledge of the topic of menstrual pain than those who did not. As the research was conducted in Canada, menstruation was likely being considered through a Western lens. The generalizability of our findings across diverse populations, including cultural and gender identities, as well as countries with lower access to mobile phones, is limited. Other evidence-based interventions, such as cognitive-behavioral therapy, were not considered in this investigation and may provide valuable alternatives to mindfulness-based approaches.

Dysmenorrhea is common in adolescence, and many adolescents are not receiving adequate treatment for their dysmenorrhea, which is problematic given the established consequences of unmanaged pain. Digital interventions that offer nonpharmacological support for dysmenorrhea may be beneficial. Our findings provide an important framework for future app development. Specific content and app feature recommendations were identified and derived directly from adolescents’ preferences and needs. Incorporation of these user recommendations in subsequent app design phases may lead to improved engagement and efficacy of digital intervention, ultimately leading to improvements in the experiences of youth with dysmenorrhea.

## Appendix

Multimedia Appendix 1 CHERRIES (Checklist for Reporting Results of Internet E-Surveys) checklist.

Multimedia Appendix 2 COREQ (Consolidated Criteria for Reporting Qualitative Research) checklist.

Multimedia Appendix 3 Proportion of participants rating each response option on a 0 to 10 scale for a question inquiring about their perception that mindfulness could be helpful for managing period pain (n=107; 0=not at all helpful and 10=extremely helpful).

## Abbreviations

CHERRIES: Checklist for Reporting Results of Internet E-Surveys
COREQ: Consolidated Criteria for Reporting Qualitative Research

## Data Availability

The data sets generated and analyzed during this study are not publicly available due to identifiable information in the data set but are available from the corresponding author on reasonable request.
