# Supplementary material for: Understanding Adolescents’ Experiences With Menstrual Pain to Inform the User-Centered Design of a Mindfulness-Based App: Mixed Methods Investigation Study
Source: JMIR Pediatr Parent. 2024 Apr 8;7:e54658. doi: 10.2196/54658 (PMC11036189; doi:10.2196/54658)
Supplement: Multimedia Appendix 3 [file pediatrics_v7i1e54658_app3.docx]

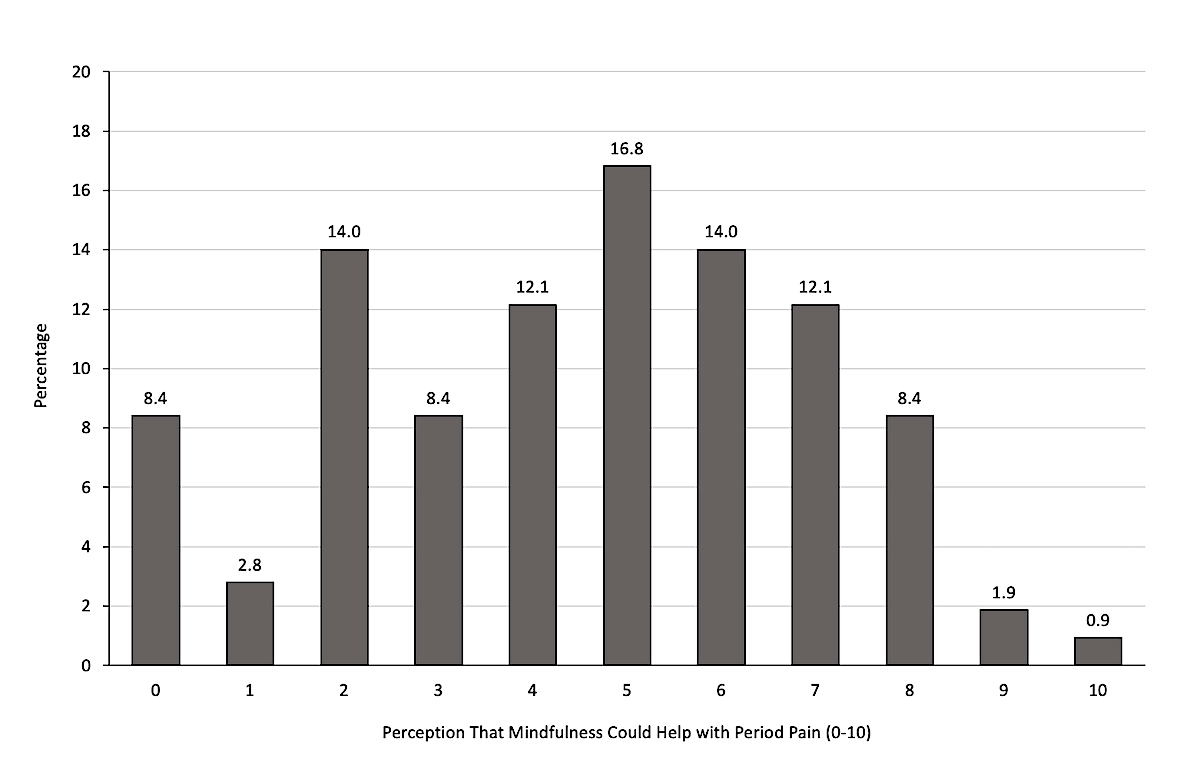


*Multimedia Appendix 3.* Proportion (%) of participants rating each response option on a 0 to 10 scale for a question inquiring about their perception that mindfulness could be helpful for managing period pain. 0 = Not at all helpful; 10 = Extremely helpful. *n* = 107.
